# Positive and negative spontaneous self-talk and performance in gymnastics: The role of contextual, personal and situational factors

**DOI:** 10.1371/journal.pone.0265809

**Published:** 2022-03-24

**Authors:** Francisco J. Santos-Rosa, Carlos Montero-Carretero, Luis Arturo Gómez-Landero, Miquel Torregrossa, Eduardo Cervelló

**Affiliations:** 1 Department of Sports and Computer Science, University Pablo de Olavide, Seville, Spain; 2 Sports Research Centre (Department of Sport Sciences), University Miguel Hernández of Elche, Elche, Spain; 3 Department of Basic, Educational and Development Psychology, University Autónoma of Barcelona, Barcelona, Spain; 4 Sport Research Institute, University Autónoma of Barcelona, Barcelona, Spain; University of Texas at San Antonio, UNITED STATES

## Abstract

The purpose of this study was to analyse whether contextual (perception of motivational climate and positive and negative spontaneous self-talk in sports), personal (positivity) and situational variables (positive and negative spontaneous self-talk employed in competition and precompetitive anxiety) predict performance in a competition of ensembles of rhythmic gymnastics. 258 female gymnasts between ages14 and 20 (*M* = 15.24, *SD* = 1.46) participated in the study, completing pre- and post-competition measures. The results of the path-analysis showed that both the task-involving climate and positivity predicted positive self-talk in sport. This predicted self-confidence which, in turn, positively predicted positive situational self-talk in competition. For its part, the perception of an ego-involving climate positively predicted the use of both negative and positive self-talk in sport. Negative self-talk in sports predicted negative situational self-talk in competition and somatic and cognitive anxiety. In turn, cognitive anxiety positively predicted negative situational self-talk. Finally, performance was positively predicted by positive situational self-talk and negatively by negative situational self-talk. These results explain the functioning of spontaneous self-talk at different levels of generality and its relationship with sports performance.

## Introduction

Recent research, both in the sport and educational areas, has highlighted self-talk as a cognitive process closely related to performance [1–3]. Self-talk is defined as “statements, phrases, or cue words that are addressed to the self which might be said automatically or strategically, either out loud or silently, phrased positively or negatively, having an instructional or motivational purpose, an element of interpretation, and incorporating some of the same grammatical features associated with everyday speech” [4, p.450].

One of the key distinctions to understand the functioning of self-talk refers to its content and function. By content, we understand the positive/negative focus of the athlete’s self-talk, which usually appears spontaneously [1]. Concerning function, we distinguish between a facilitating function and a weakening function of self-talk in performance [5].

Expressions of self-talk can have emotional content with a positive (e.g., pleasure, good mood), negative (e.g., anxiety, frustration) or neutral valence.

However, it is often not easy to distinguish between content and function because, as Hardy et al. [6] indicate, content and effect may be confused. That is, self-talk expressed as negative content could have a positive effect on performance. Therefore, to prevent this potential ambiguity between the content and functions of self-talk, these authors proposed to distinguish between positive and negative self-talk based on the content of the statements, rather than their effect.

In fact, some measuring instruments, such as the Automatic Self-Talk Questionnaire for Sport (ASTQS) by Zourbanos et al. [7] besides describing different types of self-talk oriented to different functions, group these functions into four types of positive self-talk, three types of negative self-talk and one type of neutral self-talk.

Most of the self-talk occurs as automatic and relatively effortless speech in the sports arena [8, 9]. We can distinguish two major dimensions of self-talk, depending on their nature: organic self-talk and strategic self-talk [1].

Within organic self-talk, we must also distinguish, in addition to spontaneous responses (spontaneous self-talk), athletes’ self-talk aimed at objectives, which consists of rational responses to their spontaneous responses (goal-directed self-talk). On the other hand, we also find strategic self-talk, which is a different entity, as it is not a cognitive process, but the behaviour of repeating keywords [1].

From these approaches, three research lines were developed in the study of self-talk, marked by its strategic or organic nature. The first one refers to the study of the use of programmed mental strategies in the form of routines or deliberate performance-enhancing strategies (strategic self-talk), whereas the second and third approaches refer to automatic or spontaneous self-talk, and intentional self-talk, although both approaches investigate a cognitive phenomenon [1, 3, 10].

However, researchers on self-talk also recognize that research on spontaneous organic self-talk should examine its relationships with athletic performance [1, 3]. Specifically, issues, such as how personality variables, motivational climate, or situational aspects that occur “in situ” in sports competition such as pre-competitive anxiety, and which affect self-talk should be studied for a better understanding of the mechanisms that shape content of spontaneous organic the self-talk in the prediction of athletic performance [1].

### Contextual variables. Motivational climate and self-talk

The models that study the variables that affect sport self-talk highlight that it is very sensitive to factors present in the environment [3].

Among the different theoretical models of the effect of the environment on athletes’ behavior, the Achievement Goal Theory (AGT) is one of the main theoretical frameworks for the understanding of cognitive, behavioural and emotional patterns related to achievement in sports [11, 12]. This theory states that social environments can influence individuals’ behaviours through the motivational climate to which individuals are exposed [11]. In the sporting context, the perception of a task-involving climate has been associated with more adaptive behavioural patterns for performance, whereas the perception of an ego-involving climate has been linked to maladaptive responses [13, 14].

Various works have shown that the perception of a coach-created motivational climate based on supportive behavior (emotional and esteem-building) and empowerment (task-involving climate, autonomy and social relationship supportive) was associated with greater use of positive self-talk in competition athletes [15, 16]. In contrast, a climate where negative aspects of behaviour (consisting of providing information that generates athletes’ somatic responses and negative thoughts of insecurity) and disempowerment (ego-involving climate and use of a controlling style with athletes) are perceived as related to negative self-talk [16, 17].

On the other hand, and although they are not specific to the sporting context, in the educational field, there is also evidence that autonomy-supportive climate is positively related to students’ positive self-talk and negatively to their negative self-talk [18].

In the same line, other studies have been performed that have recreated competitive situations to analyse the effect of the motivational climate on self-talk. The work of Marjanović et al. [2] showed how the perception of ego- and task-involving climates increased the use of negative and positive self-talk, respectively, highlighting the importance of teachers’ behaviour in students’ cognitive responses in Physical Education classes.

In the sports field, there is increasing evidence that coach behaviors clearly affect the use of athletes’ self-talk. In fact, some studies, like the one developed by Thibodeaux and Winsler [19], found that higher levels of perceived coach mastery climate coincided with more reported use of positive self-talk. In this line, the recent study developed by Ada et al. [20] found that the quality of coach-athlete relationships is related to the use of more positive self-talk and negatively to negative self-talk in sport. These works highlight the relevance of coach behaviors in the use of more positive organic self-talk. These results show the need to analyze the coach’s role in the use of self-talk to explain the functioning of organic sport self-talk [17].

One of the relevant contributions of this work is the study not only of how contextual variables, such as the perception of the motivational climate or the coach-athlete relationship, affect athletes’ habitual positive or negative spontaneous sport self-talk but also the analysis of the situational variables related to the frequency of spontaneous organic self-talk occurring in a specific competitive event (spontaneous self-talk "in situ" [19] or situational self-talk.

### Personal variables: Positivity and self-talk

Some works and models that study self-talk approach have shown that different personal variables affect spontaneous organic self-talk [3]. Variables such as self-esteem [21] or personality variables such as neuroticism [22] affect automatic self-talk. There is also evidence in the sports field that variables such as the anxiety trait [23], sports efficacy [24], motivational orientation [25] or self-determined motivational levels [26] affect the use of self-talk. Thus, concerning the use of positive or negative self-talk, research has shown that athletes with high motivational task orientations and moderate ego orientations tend to use positive self-talk more usually [27].

On the other hand, there are increasingly more works that try to understand how positivity affects athletes’ behavior and how they interpret the events they experience, both past and present [28].

Diener et al. [29] referred to positivity as a propensity to evaluate aspects of life in general as good. More specifically, it refers to “an individual propensity to positively evaluate or to be positively oriented toward various life domains including oneself, and one’s future and past experiences” [30, p.277]. The idea is that positivity could be part of human beings’ biological equipment that predisposes them to benefit from positive events [31]. This tendency influences positively feelings, thoughts and behaviours, thereby contributing to an overall positive individual functioning [32].

In the field of sport, positivity has been linked to optimal functioning, showing relationships with self-esteem, self-efficacy or resilience [33].

To date, no attention has been paid to the effect of positivity on self-talk, in terms of whether it can affect the frequency of more positive or negative self-talk. In this regard, if positivity provides a more optimistic view of past or present events, it would be reasonable to think that greater positivity would be related to more positive self-talk and less negative self-talk. In fact, positivity accounted for a significant portion of the variance in studies investigating people’s tendencies to rate their performance and their attributes as better than average [31]. Likewise, it would be reasonable to think that self-talk may mediate the effect of positivity on the interpretation of stressful events such as pre-competitive anxiety because some studies have shown that positivity acts negatively on stressful events like burnout, mediated by other variables such as the coping strategies for these events [34].

Therefore, it might be interesting to know the predictive nature of positivity on spontaneous organic self-talk in sport.

### Situational variables: Pre-competitive anxiety, situational self-talk and performance

Anxiety has been shown to be a key construct in understanding emotions in sports [35, 36] because it is closely related to performance [37].

In the sports context, there is evidence that the use of positive strategic self-talk (motivational and/or instructional) helps reduce levels of competitive anxiety [38, 39]. Regarding spontaneous organic self-talk, in those situations where anxiety is generated, athletes use both positive and negative self-talk [40]. In this line, the occurrence of negative self-talk has been linked to an increase in anxiety levels [41] and to different types of anxiety, such as fear of failure and sports anxiety when athletes made mistakes [23].

Another key issue in the study of self-talk is its relationship with sports performance. In the field of sport, the positive effect of strategic self-talk on sports performance is well documented, both in novice athletes [42] and in experts [43], and also in individual [39, 44] and collective sports [45, 46]. Some studies have analysed from an observational perspective the relationship between spontaneous self-talk and performance. One of them, developed by Van Raalte et al. [47], examined the effect of self-talk on the performance of 24 junior tennis players with a postmatch questionnaire. The authors found that negative self-talk was associated with losing and that players who reported believing in the utility of self-talk won more points than players who did not have such beliefs. In the same line and also in tennis, the work of Zourbanos et al. [48] found that negative verbalizations for either the server or the receiver decreased the probability of winning a game and showed that the server’s verbalizations were related to the receiver’s verbalizations and the game outcome, and vice versa.

In this line, we want to deepen the study of the relationship between spontaneous organic self-talk and performance in competition. The novelty of this work is that it contemplates variables that occur at different levels (sports in general and a competition situation) and how these variables are related to each other. At the situational level, specifically, it remains to be analysed whether the frequency of athletes’ spontaneous self-talk during a competitive situation (“situational” self-talk”) predicts their performance and whether it is affected by pre-competitive anxiety.

### Aims and hypotheses

The purpose of the present study was to examine the mechanisms underlying the antecedents, both contextual and personal, that predict how often athletes use spontaneous self-talk in sport, as well as to confirm how sports competition modifies the situational use of self-talk, ultimately affecting performance.

Specifically, the study aimed to contribute to the literature in four ways: (a) by examining the role of the perception of the motivational climate as a contextual variable and positivity, as a personality variable, in predicting the frequency of athletes’ positive and negative spontaneous self-talk in sport; (b) by analyzing how positive and negative spontaneous self-talk employed in the sports context affects how often athletes experience situational (positive and negative) spontaneous self-talk in a sports competition; (c) by verifying whether athletes’ situational self-talk can be mediated by pre-competitive anxiety; and (d) by confirming whether the frequency of athletes’ spontaneous positive and negative situational self-talk significantly predicts performance in competition.

**Fig 1:**
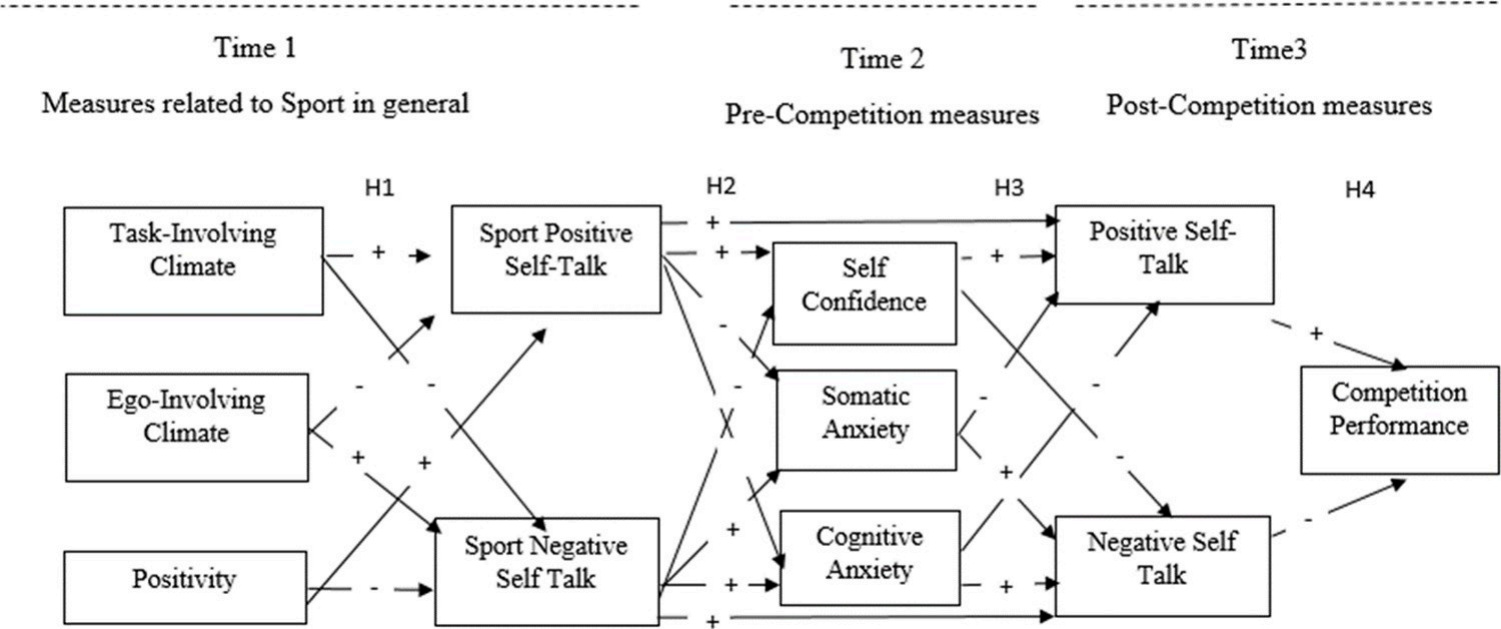
Hypothesised model.

The following specific hypotheses were formulated, based on the previous literature (Fig 1). Although different explanatory models of self-talk consider that some of these relationships may be bidirectional (e.g. [3]), given the design of this study in which the measurements are carried out at different times, we decided to exclude these bidirectional influences. They would be highly recommended if the design were longitudinal, with different measures of anxiety, performance, or situational self-talk, in which we could see if what happens at a certain time can affect what happens next. Based on this decision, our hypotheses are as follows.

First, we hypothesized that the perception of a task-involving motivational climate would positively predict how often athletes use spontaneous positive sport self-talk, and the perception of an ego-involving motivational climate would negatively predict how often athletes use spontaneous positive sport self-talk; and that the perception of a task-involving motivational climate would negatively predict how often athletes use spontaneous negative sport self-talk, and the perception of an ego-involving motivational climate would positively predict how often athletes use spontaneous negative sport self-talk (Hypothesis 1a).

We also predict that positivity would positively predict how often athletes use spontaneous positive sport self-talk and negatively predict how often they use spontaneous negative sport self-talk (Hypothesis 1b).

Second, we hypothesized that athletes’ frequency of spontaneous self-talk in sport (contextual) would predict how often they experience spontaneous self-talk in competition and pre-competitive anxiety (situational). Concretely, athletes’ frequency of positive sport self-talk would predict their frequency of positive self-talk in competition and conversely, how often athletes experience spontaneous negative self-talk would positively predict their frequency of spontaneous negative situational self-talk (Hypothesis 2a). We also hypothesized that athletes’ frequency of spontaneous positive sport self-talk would positively predict self-confidence and negatively predict somatic and cognitive anxiety. Athletes’ frequency of spontaneous negative sport self-talk would negatively predict self-confidence and positively predict somatic and cognitive anxiety (Hypothesis 2b).

At the situational level, we hypothesized that self-confidence would positively predict athletes’ frequency of spontaneous positive situational self-talk and would negatively predict their frequency of spontaneous negative situational self-talk. Somatic and cognitive anxiety would positively predict how often athletes experience spontaneous negative situational self-talk and would negatively predict their frequency of spontaneous positive situational self-talk (Hypothesis 3).

Finally, we hypothesized that athletes’ frequency of spontaneous positive situational self-talk would positively predict competition performance, and their frequency of spontaneous negative situational self-talk would negatively predict performance (Hypothesis 4).

## Method

### Participants

The study involved 258 female gymnasts from 29 different clubs. Age ranged between 14 and 20 years (*M* = 15.24, *SD* = 1.46). All of them had a minimum experience of 4 years of competitive practice in order to be part of the study. Mean experience was 5.32 years (*SD* = 1.75).

### Measuring instruments

#### Perception of motivational climate

To measure the athletes’ perception of motivational climate in sports, we employed the version translated into Spanish by [49] of the Perception of Motivational Climate in Sport Questionnaire-2 [50, 51]. The Spanish version of this questionnaire consists of two second-order dimensions that measure the perception of task-involving motivational climate, the perception of ego-involving motivational climate, and five first-order factors. For this investigation, only the second-order factors were examined, rated on 5-point Likert scales ranging from 1 (*strongly disagree*) to 5 (*strongly agree*). In the Spanish version, the task-involving climate factor is comprised of 11 items. An example of the perception of a task-involving climate is “Players feel good when they try their best”. The perception of an ego-involving climate factor includes 13 items (e.g. “The coach has his or her own favourites”). The studies carried out on Spanish athletes have shown factor patterns and internal consistency coefficients similar to those found for athletes and students in other countries [49, 52].

#### Positivity

The Positivity Scale [53] was designed as a short instrument to directly assess positivity. We used the Spanish version [33]. Items assess a positive view of oneself, one’s life and future, as well as one’s confidence in others (e.g., “I have great faith in the future”). The 8 items were rated on a 5-point Likert scales ranging from 1 *(strongly disagree*) to 5 (*strongly agree*). The instrument has shown good fit values when subjected to confirmatory factor analyses [34]. As there are not many studies that have validated the instrument in Spanish, we performed a principal components exploratory factor analysis. The results grouped the 8 items into a single factor, with factor scores between .40 and .79, explaining 47.01% of the variance. The Spanish version showed good internal consistency, with a Cronbach’s alpha of .72 [33].

#### Self-reported contextual and situational self-talk

We used the Spanish version of the Automatic Self-Talk Questionnaire for Sports [41]. This instrument measures the content and structure of the athletes´ internal self-talk in the sports context. The instrument consists of 40 items assessing four positive (19 items) and four negative (21 items) self-talk dimensions. The four positive factors are: Concentration (5 items, e.g., “Put out your best effort”), Anxiety Control (4 items, e.g., “Don’t get angry”), Confidence (5 items, e.g., “I’m well prepared”) and Instructions (5 items, e.g., “Focus on you what you have to do right now”. The negative factors are: Worry (7 items, e.g., “I can’t focus”), Retirement (5 items, e.g., “I want to drop out”), Somatic Fatigue (5 items, e.g., “My body is not fit”), and Irrelevant Thoughts (3 items, e.g., “I’m hungry”).

Some researchers have highlighted some limitations of this instrument, such as the low relationship between the measures reported by the questionnaire and athletes’ "in situ" behaviours during the competition [9]. However, in this study, the instrument showed good construct validity through high correlations between how often the athletes used positive spontaneous self-talk in competition, the perception of a mastery climate and the coach’s autonomy support [19].

Adaptations involved adapting the questionnaire to the context of gymnastics and for two different moments. (a) athletes’ frequency of spontaneous self-talk generally used during sports practice “In your sport, how often have you thought or told yourself something similar to the following ideas in the last months” and (b) athletes’ frequency of spontaneous self-talk “in situ” at a particular moment “In this competition, how often have you thought or told yourself something similar to the following ideas”. Participants indicated its frequency on a 5-point Likert scale ranging from 1 (Never) to 5 (Very often).

Similar to the study of De Muynck et al. [54], a second-order principal component analysis (PCA) with Promax rotation was developed to examine the underlying factor structure of self-reported spontaneous sport self-talk and spontaneous self-talk in competition. Two factors emerged for spontaneous sport self-talk, explaining a total of 32.89% of the variance. These factors include positive and negative spontaneous self-talk factors, with factor loadings between .77 and .41. For competition self-talk, two factors were retained, explaining in total 37.28% of the variance. These factors included clearly the positive and negative self-talk, with factor loadings ranging from 0.44 to 0.78.

Sport and competition measures of self -talk showed good internal consistence (>.70) for all factors (Table 1)

**Table 1:** Means, standard deviations, bivariate correlations, and reliability analysis of the variables.

Variables	*Rank*	*M*	*SD*	1	2	3	4	5	6	7	8	9	10	11
**1. Task-involving climate**	1–5	4.45	.47	.*78*										
**2. Ego-involving climate**	1–5	2.34	.64	-.29**	.*77*									
**3. Positivity**	1–5	3.99	.61	.26**	-.06	.*84*								
**4. Sport Positive Self-talk.**	1–5	3.61	.68	.30**	.13*	.32**	.*85*							
**5. Sport Negative Self-talk**	1–5	2.10	.68	-.13*	.35**	-.39**	-.04	.*90*						
**6. Self-Confidence**	1–4	3.48	.46	.14*	-.04	.42**	.28**	-.24**	.*78*					
**7. Somatic Anxiety**	1–4	2.51	.65	-.04	.24**	-.24**	.04	.31**	-.26**	.*81*				
**8. Cognitive anxiety**	1–4	2.60	.70	-.08	.17**	-.30**	-.07	.31**	-.29**	.47**	.*73*			
**9. Positive Situational Self-talk**	1–5	3.82	.72	.22**	.01	.20**	.65**	-.02	.24**	.17**	-.02	.*87*		
**10. Negative Situational self-talk**	1–5	1.39	.51	-.06	.17**	-.28**	-.04	.52**	-.15*	.23**	.30**	-.05	.*91*	
**11. Competition Performance**	1–5	4.01	.77	-.01	.10	.01	.16**	-.02	.13*	.02	-.07	.25**	-.25**	.*65*

*Note*.

*p < .05

**p < .01. Cronbach alpha values are on the diagonal in italics.

#### Pre-competitive anxiety

The Spanish version [55] of the Competitive Sport Anxiety Inventory 2-revised [56] was employed to measure pre-competitive anxiety. The Spanish version of the CSAI-2 R is an 18-item inventory that measures cognitive state anxiety (5 items, e.g., “I’m concerned about performing poorly”), somatic state anxiety (8 items, e.g., “I feel tense in my stomach”, “I feel jittery”) and self-confidence (5 items, e.g., “I feel self-confident”) in a competitive setting. The instrument has shown good fit values when subjected both to exploratory and confirmatory factor analyses [55, 57].

Each item on the CSAI-2R is rated on a 4-point Likert scale ranging from 1 (*not at all*) to 4 (*very much* so). As there are not many studies that have validated the instrument in Spanish, we performed a principal components exploratory factor analysis with Oblim rotation, which grouped the items into the three factors of the questionnaire. The factors explained 51.06% of the variance. The factor loadings ranged between .79 and .45.

The Spanish version of the instrument has shown good levels of internal reliability (alpha coefficients for the factors ranged from .79 to .83).

#### Performance

Considering that, in gymnastics, the team’s score in the competition can lead to ambiguity about individual performance, we considered both the subjective perception of each gymnast of their performance and the coach’s perception in order to measure performance. Some researchers have recommended that, when a psychological state of interest is related to a performance outcome, two independent assessments of performance outcome should be used, reflecting both the athlete’s and coach’s assessment [52, 58, 59]. Therefore, the coach was asked to respond to the following statement, “Considering this gymnast’s usual performance, rate how you perceived her performance in today’s competition”. The gymnasts also responded to this statement about their personal performance. We measured the two statements in the form of a Likert scale ranging from 1 (*performed much worse than usual*) to 5 (*performed much better than usual*). The players’ self-assessments and coaches’ assessments were completed immediately after the game. As the two measures showed a moderate and significant correlation (*r* = .49, *p* < .001) and the alpha of the two items was .65, we used a composite of the two measures for statistical analysis.

### Procedure

This research was supported by a research project from the first author´s university and the Andalusian Gymnastics Federation. The study received the ethical approval of the university (2019/00183/001). All participants were treated according to the American Psychological Association’s ethical guidelines regarding consent, confidentiality, and anonymity of responses. Informed consents were sent to the parents/guardians of underage gymnasts and their acceptance was an essential condition to participate in the study. For data collection, the gymnasts were informed about the object of the study, how to complete the questionnaire, and the importance of their sincerity in the answers. A cross-sectional design was used.

The sample was collected in three stages: The first took place during a training week, without competition, where the personal (positivity) and contextual variables (perception of motivational climate and self-talk in sport) were measured (Time 1); the second and third phases were conducted during a tournament, qualifying for the final phase of the National Championship for Teams. Pre- and post-competition measurements were collected. The pre-competition measure (pre-competitive anxiety) was collected 15 to 20 minutes before the competition (Time 2). The post-competition measure (situational self-talk and performance) was done 5 minutes after the competition (Time 3).

### Data analysis

To explore the relationship of contextual and personal variables (perception of positivity, motivational climate) self-talk in sport, and the competition variables (pre-competitive anxiety, competition self-talk and performance), firstly, a descriptive and correlational analysis was carried out. The reliability of the instruments measuring these variables was tested through Cronbach’s alpha.

Also, given that the objective of the work was to test whether the contextual, personal and sport self-talk variables predicted the behavior of competition self-talk and whether this, in turn, predicted performance, a path-analysis was used to study the sequential temporal model presented in the hypotheses (Fig 1). For the analysis, the Amos 26 of IBM SPSS software was used. The exploration of model fit indices followed the guidelines of [60], considering the following goodness-of-fit indices: chi-square/*df* values between 2 to 3, with limits of up to 5; incremental fit indices greater than .90; and error fit indices of less than .08 for the root mean square error of approximation (RMSEA) and less than .06 for the standardised root mean square residual (SRMR). Hu and Bentler [60] recommend contemplating several of these indices to accept or reject a model. To improve the overall fit, error variances were correlated between dimensions based on theoretical relationships (i.e., dimensions under one construct) and statistical evidence (i.e., modification indices > 10, [61]).

## Results

### Descriptive statistics and correlations

Table 1 shows rank, means, standard deviations, reliability statistics and correlations of the analysed variables. All scales had acceptable internal consistency (i.e., >.70), the performance measure (*α* = .65) was also accepted as valid although it only had 2 items [62]. The means of the factors showed higher levels of perception of task-involving climate *(M* = 4.45, *SD* = 0.47) and moderate levels t of ego-involving climate *(M* = 2.34, *SD* = 0.64).

Regarding the frequency of self-talk, we found high levels of frequency of both contextual and situational positive self-talk (*M* = 3.61, *SD* = 0.68 and *M* = 3.82, *SD* = 0.72, respectively). Contextual and situational negative self-talk was moderate (*M* = 2.10, *SD* = 0.68 and *M* = 1.39, *SD* = 0.51, respectively). Self-confidence levels *(M* = 3.48, *SD* = 0.46), were high, and somatic anxiety (*M* = 2.51, *SD* = 0.65) and cognitive anxiety (*M* = 2.60, *SD* = 0.70) perceived before competition were moderate.

Both perceived performance *(M* = 4.01, *SD* = 0.77), and positivity *(M* = 3.99, *SD* = 0.61*)* were high, taking into account the response range of these variables (1–5).

The correlation analyses yielded positive relations between positive sport self-talk and task-involving climate (*r* = .30, *p* < .01), positivity (*r* = .32, *p* < .01), positive situational self-talk (*r* = .65, *p* < .01), self-confidence (*r* = .28, *p* < .01) and performance in competition (*r* = .16, *p* < .01). Negative sport self-talk was significantly and positively related to ego-involving climate (*r* = .35, *p* < .01), negative situational self-talk (*r* = .52, *p* < .01), somatic anxiety (*r* = .31, *p* < .01) and cognitive anxiety (*r* = .31, *p* < .01), and negatively related to self-confidence (*r* = -.24, *p* < .01).

With regard to situational self-talk, the results show that positive self-talk was significantly and positively related to somatic anxiety (*r* = .17, *p* < .01), self-confidence (*r* = .24, *p* < .01) and performance in competition (*r* = .25, *p* < .01), whereas negative self-talk showed a significant and positive relationship with somatic anxiety (*r* = .23, *p* < .01) and cognitive anxiety (*r* = .30, *p* < .01), and a negative relationship with self-confidence (*r* = -.15, *p* < .05) and performance (*r* = -.25, *p* < .01).

### Path analysis

A path analysis was conducted, analysing the fit of the hypothesised sequential model presented (Fig 1) considering the motivational climate, positivity, sport self-talk, pre-competitive anxiety, situational self-talk, and performance. To estimate the path-analysis model, the Maximum Likelihood (ML) method was used, suitable for our model because the assessment of normality revealed that all the variables were sufficiently normally distributed. All skewness and kurtosis values were under 3 and 10, respectively, as [63] recommends as a cut-off. The distribution was also found to be normal at the multivariate level. The Mardia coefficient obtained a value of 15.19, below the minimum value (< 70), which indicates multivariate normality [60].

The model posed in our hypotheses was tested. The goodness-of-fit indices showed an unsatisfactory fit of the model, χ^2^(30) = 128.317, CFI = .842, TLI = .710, AIC = 222.31, RMSEA = .113, 95% CI [.093, .133], SRMR = .079. The modification indices were examined. The errors between dimensions of pre-competitive anxiety showed a modification index above 10, so these error variances were correlated. The modified model showed good fit indices both for incremental and error indices, χ^2^(27) = 57.32, CFI = .951, TLI = .900, AIC = 157.32, RMSEA = .066, 95% CI [.042, .090], SRMR = .053.

**Fig 2:**
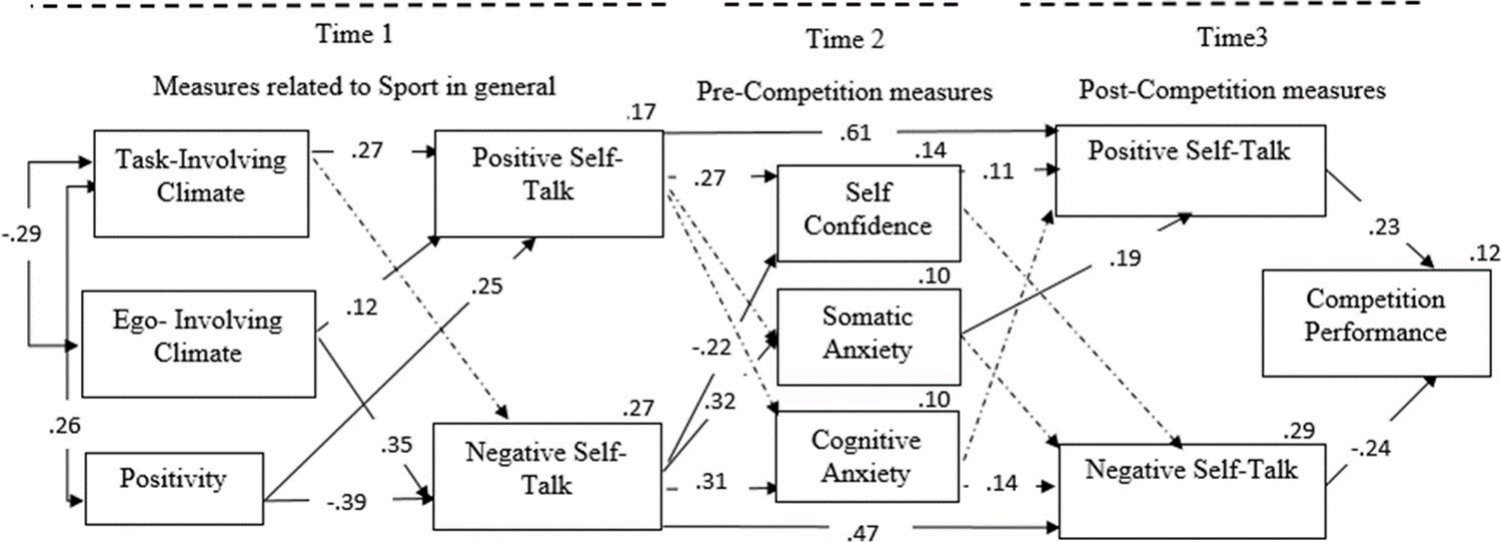
Final model with standardised parameter estimates. Significant paths are represented with solid lines and non-significant paths are shown with dotted lines.

The final model is shown in Fig 2, with the significant paths represented with solid lines and the non-significant ones with dashed lines. The results showed that the perception of a task-involving motivational climate (β = .273, *p* < .001) and positivity (β = .261, p < .001) positively predicted positive sport self-talk. Positivity also negatively predicted negative sport self-talk (β  =  -.391, *p* < .001). The perception of an ego-involving motivational climate positively predicted negative sport self-talk (β = .350, *p* < .001) and positive sport self-talk (β  =  .128, *p* = .032). Direct effects also showed that positive sport self-talk predicted positive situational self-talk (β = .479, *p* < .001) and that negative sport self-talk predicted negative situational self-talk (β  =  .612, *p* < .001). The results showed that positive sport self-talk positively predicted self-confidence (β  =  .272, *p* < .001), and negative sport self-talk, positively predicted pre-competitive somatic anxiety (β  =  .320, *p* < .001), cognitive anxiety (β  =  .312, *p* < .001), and negatively predicted self-confidence (β  =  -.229, *p <* .001). Fig 2 also shows that self-confidence (β  =  .116, *p* = .022) and somatic anxiety (β  =  .195, *p* < .001), positively predicted the use of positive situational self-talk, and cognitive anxiety positively predicted the use of negative situational self-talk (β  =  .147, *p* = .017). Analyzing performance, positive situational self-talk positively predicted performance (β  =  .238, *p* < .001), and negative self-talk negatively predicted performance (β  =  -.242, *p* < .001).

## Discussion

The objective of this work was to show, for the first time in the field of sport, the simultaneous interactions occurring between different contextual, personal and situational factors related to self-talk. This study can help us gain a better understanding of how self-talk works, both in sport in general and in a particular competitive situation.

Regarding the relationship between the perception of the motivational climate and the use of sport self-talk (H1a), the results have partially corroborated our hypothesis. Specifically, we found that the perception of a task-involving climate positively predicted positive self-talk in sport, whereas the perception of an ego-involving climate predicted negative self-talk in sport. These results are in line with the works analysing the relationship between contextual factors and self-talk, which have found that the perception of task-involving climates and coach empowerment behaviour increased the use of positive self-talk [2, 15, 16]. An unexpected and noteworthy result is that also the perception of an ego-involving climate positively predicted positive self-talk in sport. Whereas the weight of the prediction is not very high, it would be interesting to check whether this result is a feature of our sample or a common issue in athletes with competition experience. We note that our athletes are rhythmic gymnasts, performance-oriented, and with several years of experience at a competitive level. When studying athletes with competitive experience, there is evidence that an ego-involving climate can be adaptive to improve some performance-related variables, such as flow [64].

From a practical perspective, it should be noted that these results imply that, in highly ego-oriented athletes, coaches should not always try to avoid this type of orientation but rather seek a balance with organic self-talk aimed at objectives, which involves a cognitive process of reflection.

Regarding the study of the relationship between the personal factor positivity and self-talk (H1b), we hypothesised that positivity would positively predict the use of positive sport self-talk, and negatively would predict the use of negative sport self-talk. Our results have confirmed that, indeed, positivity is a personality factor that significantly predicts both the use of positive (positively) and negative self-talk (negatively). Previous studies have shown how personality affects self-talk. In fact, an opposite factor to the one which we analysed, pessimism, has been shown to be a predictor of greater use of negative self-talk [65], in the line of our results.

Regarding our second hypothesis, we had expected to find, on the one hand, that the usual self-talk used in sports would predict the situational self-talk employed in a sports competition (H2a). The results corroborate this hypothesis and show that the tendency to use one type or another of self-talk in the sports context very powerfully predicts situational self-talk. One could consider that both kinds of self-talk are collinear, that is, that it is actually the same variable at different times. To check this issue at the statistical level, we decided to calculate a linear regression including performance as a dependent variable and the four types of self-talk (contextual and situational) as independent predictor factors. The results showed that situational self-talk was the best predictor of performance. In addition, the calculated collinearity statistics showed that the tolerance values between the contextual and situational self-talk ranged from .52 to .72, and the Variance Inflation Factors (VIF) ranged from 1.38 to 1.75, indicating the absence of collinearity [66] between the contextual and situational dimensions of self-talk. These data seem to support that self-talk is also structured at different levels of generality (contextual and situational) and that, although there is a tendency to contextually use one type or another of self-talk, there are also situational variables that seem to modulate this tendency to use self-talk situationally, as we will see in the discussion of our third working hypothesis.

In our second hypothesis, we also predicted that the use of positive or negative self-talk would cause athletes to experience higher or lower levels of anxiety before competition (H2b). We believe that this approach is one of the most relevant contributions of our work, as the perspective from which the relationship between self-talk and anxiogenic or stressful situations in sports has traditionally been analysed is the effect of these situations on self-talk [23, 40, 41]. From another perspective, we analysed whether contextual self-talk makes athletes interpret anxious situations differently. This approach is not new and was already used in the work of Chen and Hardy [67], in which they analysed both the function (instructional/motivational) and the content (positive/negative) of self-talk in the prediction of anxiety levels in a sample of Chinese athletes and dancers. In this study, they found that negative self-talk positively predicted both somatic and cognitive anxiety. Our results are along the same lines as this work, as we found that positive self-talk positively predicted self-confidence, whereas negative self-talk predicted somatic and cognitive anxiety and significantly reduced the gymnasts’ self-confidence before a competition. These results show that the perception of an anxious situation varies depending on the type of self-talk commonly used in sports. However, more research is necessary to study more thoroughly the relationship between contextual and situational variables, which allows us to determine more clearly how self-talk functions in sports to predict athletes’ pre-competitive anxiety levels.

At the competition level, we hypothesised that the anxiety experienced before a competition will predict the self-talk used during the competition (H3). The results have partially verified the hypothesis, as we found that self-confidence positively predicted positive self-talk in competition. This result supports the relevance of self-confidence in the development of adaptive performance patterns and coincides with the results found by [68], who, when relating pre-competitive anxiety and performance in artistic gymnasts, found that only self-confidence differentiated between high- and low-performing groups.

Our results have also shown that cognitive anxiety directly predicted negative self-talk in competition, but contrary to our hypotheses, somatic anxiety also positively predicted positive self-talk in sport. This latter result could mean that certain pre-competition somatic alert levels seem to stimulate positive self-talk. In future work, it would be interesting to see whether the interpretation of somatic symptoms of anxiety facilitates [35] the effect of somatic anxiety on the increase of positive self-talk in competition. Referring to anxiety and self-talk, in this line, the work developed by [40] found as the main result that, in situations that generated anxiety, athletes tended to use both positive and negative anticipatory spontaneous self-talk to a greater extent.

Recently, the relevance of self-talk in emotion regulation in competition has been studied in depth, analysing how emotions are regulated by situational self-talk. Thus, the work developed by [69] found that goal-directed self-talk is a more powerful regulator of intense negative emotions than spontaneous self-talk in competition. They also found that emotion regulation is more powerful when both types of self-talk are used, which demonstrates the bidirectional effect, not only of anxiety on self-talk but also of self-talk on the reduction of anxiety.

Within the study of competition variables, our last hypothesis (H4) stated that positive situational self-talk would positively predict competition performance, and negative situational self-talk would negatively predict performance. The results have fully corroborated this hypothesis, as we found that positive self-talk positively predicted performance, whereas negative self-talk did so negatively. Although it is already well documented that strategic self-talk improves strategic performance, this work is one of the first to study the function of spontaneous self-talk in the prediction of performance in a competitive situation, and it has shown the relevance of spontaneous self-talk in predicting athletic performance.

### Strengths, limitations, and future research

We believe one of the strengths of our work has been to contemplate a global vision of the functioning of spontaneous self-talk, considering social and personal variables as well as different levels of generality (contextual and situational) of self-talk. Measuring the behavior of these variables in training and competition situations, and in an ecological competitive environment, allows exploring the relationships required by research to understand the interactions between personal, contextual and situational variables related to spontaneous self-talk.

One limitation, common to some works that measure self-talk, is the use of ex-post measures. As has already been commented by other researchers, this has advantages and disadvantages [5]. We have tried to minimize the disadvantage of the time effect (and therefore not depend on the athletes’ recall capacity) between the use of the situational self-talk and its measurement. For this purpose, we measured it just after the sporting competition. New studies should take this issue into account when designing data-taking procedures.

On the other hand, the results of our research lead to several future research prospects. Concerning the relationship between personality variables and self-talk, it would be interesting to study how other personality variables affect the use of different types of self-talk and to verify this effect in competitive situations that are ecologically valid. Specifically, it would be interesting to determine whether variables such as resilience [70] are related to the maintenance of positive self-talk in situations of competitive adversity or with high levels of anxiety.

Another issue that should be deepened in future research is whether self-talk is structured at different levels of generality, as has already been studied and demonstrated in other psychological processes such as self-determined motivation [71]. If so, this would open up a new field of study of the influences between the different levels of generality and help to establish the methodological guidelines for the design of intervention programs for the improvement of self-talk.

For example, in this work, at the situational level, we studied how pre-competitive anxiety affects self-talk in competition. It would be interesting to incorporate new situational variables that might modulate competitive self-talk. Some of the most relevant would be the effect of the motivational climate that created by the coach during sports competition [52], or how the directionality of anxiety [35] and not only its intensity—as analysed in our study—affects the use of spontaneous self-talk in competition.

Another issue that remains to be analyzed, as mentioned in the hypotheses, is to study some bidirectional relationships between variables. New studies could examine, for example, the predictive power of positivity in automatic sport self-talk, and determine, for example, how positivity is expressed through automatic sport self-talk.

## Conclusions

The results obtained in this work have clear practical consequences. The results have shown the need to inform and train coaches in the importance of the personal and contextual keys in the emergence of positive or negative self-talk in their athletes. It is also necessary for training programs on self-talk to take into account the results of this work, both in terms of athletes’ personal characteristics and situational requirements, so that more successful interventions regarding performance can be designed.

In conclusion, we believe that this work has presented a holistic view of the interactions between personal, contextual and situational factors that relate to self-talk. The results have highlighted the relevance of the work of coaches in the development of more adaptive self-talk, through the promotion of task-involving climates, the importance of positivity as a key factor in the prediction of self-talk, and the effects of situational variables on the use of self-talk in competition and its relationship with sports performance. All this opens the door to new works that examine in greater depth the mechanisms presented here and their practical implications.
